# Age-Related Clinical Patterns and Presentations of Tinea Versicolor: A Cross-Sectional Study

**DOI:** 10.7759/cureus.97091

**Published:** 2025-11-17

**Authors:** Abdullah Abdullah, Azhar Abttan

**Affiliations:** 1 General Medicine, Barts Health NHS Trust, London, GBR; 2 Dermatology, Al Karamah Teaching Hospital, Baghdad, IRQ

**Keywords:** acne vulgaris, age, clinical patterns, distribution, hyperpigmentation, hypopigmentation, malassezia, pediatric dermatology, pityriasis versicolor, tinea versicolor

## Abstract

Background: Tinea versicolor (TV) is a common superficial fungal infection caused by *Malassezia *species. While generally considered a condition of young adults, TV can affect individuals across a wide age spectrum. However, age-specific clinical presentations and patterns have not been well characterized in the literature. Understanding how TV presents across different age groups could inform clinical diagnosis and management strategies.

Objective: This study aimed to characterize the clinical presentations of TV across different age groups and identify age-related patterns in lesion type, distribution, and associated factors.

Methods: A hospital-based cross-sectional study was conducted at the dermatology outpatient clinic of Al Karamah Teaching Hospital, from June 2019 to February 2020. Consecutive patients diagnosed with TV were enrolled. Data on age, clinical characteristics (lesion type, anatomical distribution, skin type), and comorbidities were collected. Patients were categorized into three age groups: <20 years (pediatric/adolescent), 20-30 years (young adult), and >30 years (older adult). Chi-square test and Pearson correlation were used for statistical analysis.

Results: A total of 70 patients with TV were enrolled, with ages ranging from eight to 62 years (mean: 27.6 ± 11.4 years). The distribution across age groups was: <20 years (28.6%, n=20), 20-30 years (42.9%, n=30), and >30 years (28.6%, n=20). Hyperpigmented lesions were more common in younger patients (<20 years: 70.0%) compared to older adults (>30 years: 35.0%, p=0.03). Conversely, hypopigmented lesions increased with age (<20 years: 25.0% vs. >30 years: 60.0%, p=0.02). Pediatric/adolescent patients (<20 years) more frequently presented with facial involvement (35.0% vs. 13.3% in young adults and 10.0% in older adults, p=0.04). The prevalence of greasy/seborrheic skin decreased with age (70.0% in <20 years vs. 40.0% in >30 years, p=0.04). Comorbidities, particularly diabetes mellitus, were significantly more common in older patients (85.0% in >30 years vs. 5.0% in <20 years, p<0.001), while younger patients showed higher rates of concurrent acne vulgaris (70.0% vs. 10.0%, p<0.001).

Conclusions: TV demonstrates distinct age-related clinical patterns, with younger patients more likely to present with hyperpigmented lesions, facial involvement, and seborrheic skin, while older patients show predominantly hypopigmented lesions and a higher comorbidity burden. These findings suggest that age-specific diagnostic and management approaches may be beneficial. The identification of TV in pediatric patients should prompt evaluation for acne, while TV in older adults warrants screening for metabolic conditions. This study contributes important data on pediatric TV presentation, an understudied area in dermatology literature.

## Introduction

Tinea versicolor (TV), also known as pityriasis versicolor, is a common superficial fungal infection of the skin caused by lipophilic yeasts of the *Malassezia *genus, particularly *M. globosa*, *M. furfur*, and *M. sympodialis* [1]. The condition is characterized by hyperpigmented or hypopigmented scaly macules and patches, most commonly affecting the trunk, neck, and proximal extremities [2]. The term “versicolor” derives from the variable colors that the lesions can display, including white, pink, tan, brown, or salmon-colored patches [3].

While TV is traditionally described as a condition predominantly affecting adolescents and young adults, it can occur across a wide age spectrum from childhood through late adulthood [4]. The prevalence of TV varies geographically, affecting approximately 1% of individuals in temperate climates but up to 40% in tropical and subtropical regions [5]. The condition’s occurrence is closely linked to factors that promote *Malassezia *overgrowth, including increased sebum production, hot and humid environments, hyperhidrosis, and immunosuppression [6].

Age-related factors significantly influence skin physiology and may consequently affect TV presentation. Sebaceous gland activity increases during puberty under androgenic stimulation and remains elevated through young adulthood before gradually declining with age [7]. Since *Malassezia *species are lipophilic organisms that thrive in sebum-rich environments, age-related changes in sebum production could theoretically influence both TV susceptibility and clinical presentation [8]. Additionally, immune system changes across the lifespan, variations in skin pigmentation responses, and age-specific comorbidities may all contribute to different clinical patterns of TV [9].

Despite TV being one of the most common dermatological conditions worldwide, there is limited systematic data on how the condition presents across different age groups. Most existing literature focuses on young adults, with relatively sparse information on pediatric presentations and older adult cases [10]. Understanding age-specific patterns of TV could have important clinical implications, including improved diagnostic accuracy, age-appropriate management strategies, and identification of age-specific risk factors or comorbidities.

Pediatric TV, in particular, has received limited attention in the dermatological literature. While case reports exist, systematic studies characterizing the clinical features of TV in children and adolescents are scarce [11]. Given that this age group experiences significant hormonal changes during puberty, has active sebaceous glands, and may have different immune responses compared to adults, pediatric TV may present with distinct characteristics that warrant specific attention [12].

Therefore, this study aimed to characterize the clinical presentations of TV across different age groups, identify age-related patterns in lesion characteristics and distribution, and examine the association between age and concurrent dermatological or systemic conditions. We hypothesized that younger patients would more commonly present with hyperpigmented lesions and seborrheic skin, while older patients would show different clinical patterns potentially influenced by age-related physiological changes.

## Materials and methods

Study design and setting

This was a hospital-based cross-sectional observational study conducted at the dermatology outpatient clinic of Al Karamah Teaching Hospital (in Baghdad, Iraq) from June 2019 to February 2020.

Study population and sampling

The study included consecutive patients presenting to the dermatology clinic who received a clinical diagnosis of TV during the study period. Patients of all ages who met the diagnostic criteria and provided consent (or parental consent for minors) were included. Patients who declined participation or had incomplete clinical data were excluded.

Inclusion and exclusion criteria

Inclusion criteria were: (i) clinical diagnosis of TV by experienced dermatologists based on characteristic findings; (ii) patients of all ages presenting to the dermatology clinic during the study period; (iii) availability of complete clinical data including age, lesion characteristics, anatomical distribution, and skin type assessment; and (iv) informed consent obtained from adult patients or parental consent for minors.

Exclusion criteria were: (i) patients who declined to participate in the study; (ii) incomplete clinical data or missing key variables; (iii) patients with concomitant skin conditions that could confound the assessment of TV lesions; and (iv) patients who had received treatment for TV within the previous four weeks that could alter clinical presentation.

Diagnostic criteria

TV was diagnosed clinically based on characteristic findings, including hyperpigmented or hypopigmented scaly macules or patches, a typical distribution pattern (predominantly trunk, neck, upper extremities), characteristic fine scale (“cigarette paper” scale), and clinical appearance consistent with TV. The diagnosis was made by experienced dermatologists. While potassium hydroxide (KOH) examination and Wood’s lamp evaluation are confirmatory tests, the diagnosis was primarily clinical, given the characteristic presentation in most cases.

Data collection

A structured data collection form was designed to systematically record the following information. Demographic data included age (in years) and gender. Clinical characteristics of TV documented were the type of lesions (hyperpigmented, hypopigmented, or mixed), anatomical distribution (face, neck, chest/upper trunk, back, shoulders, upper arms, abdomen, lower trunk), extent of involvement (localized vs. widespread), and symptoms (presence of itching, reported recurrence). Skin characteristics were assessed by clinical examination and classified as greasy/seborrheic (characterized by visible sebum on skin surface, oily appearance, and prominent pores, particularly on face and upper trunk), normal (balanced hydration without excessive oiliness or dryness), or dry (rough texture, reduced sebum production, and scaling). Extent of involvement was classified as localized (affecting 1-2 anatomical sites) or widespread (affecting three or more anatomical sites). All assessments were performed by experienced dermatologists using standardized clinical examination protocols.

Associated conditions recorded included the presence of acne vulgaris (current active lesions), personal history of diabetes mellitus, personal history of anemia or other relevant comorbidities, and family history of TV, acne, and diabetes mellitus.

Age group classification

For analytical purposes, patients were categorized into three age groups based on physiological and clinical considerations. The <20 years (pediatric/adolescent group) included children and adolescents with active pubertal changes and developing sebaceous gland activity. The 20-30 years (young adult group) represented peak sebaceous activity and the traditional high-risk age for TV. The >30 years (older adult group) represented declining sebaceous activity and increased likelihood of age-related comorbidities.

Variables

The primary outcome variables were: (i) type of TV lesions (hyperpigmented, hypopigmented, or mixed), (ii) anatomical distribution patterns of lesions, and (iii) skin type (greasy/seborrheic, normal, or dry). 

The independent variable was age, analyzed both as a continuous variable and categorized into three groups: <20 years (pediatric/adolescent), 20-30 years (young adult), and >30 years (older adult).

Secondary outcome variables included: (i) presence of concurrent acne vulgaris, (ii) comorbidities (diabetes mellitus, anemia), and (iii) family history of TV, acne, and diabetes mellitus.

Statistical analysis

Data were analyzed using IBM SPSS Statistics version 26.0 (IBM Corp., Armonk, NY, USA). Descriptive statistics included means ± standard deviation (SD), median, and range for continuous variables and frequencies with percentages for categorical variables. Chi-square test or Fisher’s exact test was used to compare categorical variables across age groups. Pearson correlation was used to assess relationships between age as a continuous variable and ordinal outcomes. A p-value <0.05 was considered statistically significant.

Ethical considerations

This retrospective observational study was conducted in accordance with the Declaration of Helsinki principles and local institutional guidelines for clinical research. In accordance with institutional policy at Al Karamah Teaching Hospital for retrospective chart reviews using de-identified data, formal institutional review board (IRB) approval was not required for this study. All patient data were anonymized prior to analysis, and patient confidentiality was maintained throughout the study. Clinical data were collected as part of routine clinical care, and no experimental interventions were performed. No individual patients can be identified from the study results.

## Results

Overall demographics

A total of 70 patients with clinically diagnosed TV were enrolled during the study period. The age range was eight to 62 years, with a mean age of 27.6 ± 11.4 years and a median age of 23 years. The age distribution showed that TV affected individuals across a broad spectrum, with representation in pediatric (youngest: eight years), adolescent, young adult, and older adult populations (oldest: 62 years).

Age group distribution

When categorized into three age groups, the distribution was relatively balanced, with 20 patients (28.6%) in the <20 years pediatric/adolescent group, 30 patients (42.9%) in the 20-30 years young adult group, and 20 patients (28.6%) in the >30 years older adult group. The young adult group (20-30 years) represented the largest proportion, consistent with literature describing TV as primarily affecting this age range. However, the substantial representation of both younger (<20 years) and older (>30 years) patients highlights that TV is not exclusively a condition of young adults.

Age and lesion type

Significant age-related differences were observed in the type of TV lesions (Table 1). Hyperpigmented lesions were significantly more common in younger patients, present in 70.0% (14/20) of those <20 years, 56.7% (17/30) of the 20-30 year group, and only 35.0% (7/20) of those >30 years (p=0.03 for trend).

**Table 1:** Type of tinea versicolor lesions by age group P-values were calculated using chi-square test. Percentages represent the proportion of patients within each age group presenting with each lesion type.

Lesion type	<20 years (n=20)	20-30 years (n=30)	>30 years (n=20)	P-value
Hyperpigmented	14 (70.0%)	17 (56.7%)	7 (35.0%)	0.03
Hypopigmented	5 (25.0%)	11 (36.7%)	12 (60.0%)	0.02
Mixed	1 (5.0%)	2 (6.7%)	1 (5.0%)	0.95

Conversely, hypopigmented lesions showed an inverse relationship with age, being least common in the youngest group (25.0%, 5/20) and most common in the oldest group (60.0%, 12/20), with the 20-30 year group showing intermediate prevalence (36.7%, 11/30) (p=0.02 for trend).

Mixed-type lesions (both hyperpigmented and hypopigmented) were uncommon overall, present in only four patients (5.7%), with no significant age-related pattern.

Age and anatomical distribution

Anatomical site involvement showed notable age-related patterns (Table 2). Facial involvement was significantly more common in the pediatric/adolescent group, affecting 35.0% (7/20) of patients <20 years compared to 13.3% (4/30) in the 20-30 year group and 10.0% (2/20) in those >30 years (p=0.04).

**Table 2:** Anatomical distribution by age group P-values were calculated using chi-square test. Percentages represent the proportion of patients within each age group with involvement of each anatomical site.

Site	<20 years (n=20)	20-30 years (n=30)	>30 years (n=20)	P-value
Face	7 (35.0%)	4 (13.3%)	2 (10.0%)	0.04
Neck	9 (45.0%)	6 (20.0%)	5 (25.0%)	0.11
Chest/upper trunk	15 (75.0%)	22 (73.3%)	15 (75.0%)	0.68
Back	6 (30.0%)	10 (33.3%)	6 (30.0%)	0.85
Shoulders	5 (25.0%)	6 (20.0%)	4 (20.0%)	0.86
Upper arms	4 (20.0%)	5 (16.7%)	3 (15.0%)	0.90

The chest/upper trunk was the most commonly affected site across all age groups (range: 70-80%), with no significant age-related differences (p=0.68). Back involvement was also common across all groups (28-35%, p=0.85).

Neck involvement showed a trend toward higher prevalence in younger patients (45.0% in <20 years vs. 20.0% in 20-30 years vs. 25.0% in >30 years), but this did not reach statistical significance (p=0.11).

Age and skin type

The prevalence of greasy or seborrheic skin showed a significant inverse relationship with age (p=0.04). In the <20 years group, 70.0% (14/20) had greasy/seborrheic skin compared to 56.7% (17/30) in the 20-30 years group and 40.0% (8/20) in the >30 years group (Table 3).

**Table 3:** Skin type distribution by age group P-values were calculated using chi-square test. Percentages represent the proportion of patients within each age group with each skin type.

Skin type	<20 years (n=20)	20-30 years (n=30)	>30 years (n=20)	P-value
Greasy/seborrheic	14 (70.0%)	17 (56.7%)	8 (40.0%)	0.04
Normal	6 (30.0%)	12 (40.0%)	11 (55.0%)	-
Dry	0 (0%)	1 (3.3%)	1 (5.0%)	-

This finding aligns with known physiological patterns of sebaceous gland activity, which peaks during adolescence and young adulthood and gradually declines thereafter.

Age and concurrent acne vulgaris

A striking age-related pattern was observed for concurrent acne vulgaris (p<0.001). Among patients <20 years, 70.0% (14/20) had active acne, compared to 36.7% (11/30) in the 20-30 year group and only 10.0% (2/20) in those >30 years.

This strong association likely reflects the shared pathophysiological factors between TV and acne, both of which are associated with increased sebaceous activity during adolescence and young adulthood.

Age and comorbidities

Significant age-related differences were observed in comorbidity patterns (Table 4). Diabetes mellitus showed a dramatic increase with age, present in only 5.0% (1/20) of patients <20 years, 16.7% (5/30) of the 20-30 year group, and 85.0% (17/20) of those >30 years (p<0.001).

**Table 4:** Comorbidities by age group P-values were calculated using chi-square test. Percentages represent the proportion of patients within each age group with each comorbidity or associated condition. DM: diabetes mellitus; TV: tinea versicolor

Comorbidity	<20 years (n=20)	20-30 years (n=30)	>30 years (n=20)	P-value
Concurrent acne	14 (70.0%)	11 (36.7%)	2 (10.0%)	<0.001
Personal history of DM	1 (5.0%)	5 (16.7%)	17 (85.0%)	<0.001
Personal history of anemia	2 (10.0%)	4 (13.3%)	2 (10.0%)	0.89
Family history of DM	1 (5.0%)	6 (20.0%)	9 (45.0%)	0.004
Family history of acne	2 (10.0%)	3 (10.0%)	2 (10.0%)	1.00
Family history of TV	1 (5.0%)	4 (13.3%)	2 (10.0%)	0.63

Personal history of anemia showed no significant age-related pattern (10.0% in <20 years, 13.3% in 20-30 years, and 10.0% in >30 years, p=0.89).

Family history of diabetes mellitus was more common in the older age groups (5.0% in <20 years vs. 20.0% in 20-30 years vs. 45.0% in >30 years, p=0.004), which may reflect both genetic predisposition and age-related expression of diabetes in family members.

Correlation analysis

When age was analyzed as a continuous variable, significant correlations were found with several clinical features. Age showed a positive correlation with hypopigmented lesions (r=0.31, p=0.009) and a negative correlation with hyperpigmented lesions (r=-0.29, p=0.014). Additionally, age demonstrated negative correlations with greasy skin (r=-0.25, p=0.037) and concurrent acne (r=-0.51, p<0.001), indicating these features were more common in younger patients. The strongest correlation was observed between age and diabetes mellitus (r=0.71, p<0.001), reflecting the higher prevalence of diabetes in older patients.

Pediatric subgroup analysis

Given the limited literature on pediatric TV, we performed additional descriptive analysis of the youngest patients. Among the 20 patients <20 years (range: 8-19 years), the mean age was 15.8 ± 3.2 years. The youngest patient was eight years old with hyperpigmented lesions on the face and upper trunk. In this pediatric/adolescent subgroup, 85.0% (17/20) had either greasy skin or concurrent acne, or both, while only 15.0% (3/20) had no sebaceous-related findings. Facial involvement was present in 35.0% (7/20), higher than in any other age group. No patients had diabetes mellitus except one 16-year-old with type 1 diabetes.

## Discussion

This cross-sectional study of 70 patients with TV demonstrates clear age-related patterns in clinical presentation, with significant differences in lesion type, anatomical distribution, skin type, and associated conditions across age groups. These findings have important implications for clinical diagnosis, management, and understanding of TV pathophysiology.

Lesion type and age

One of the most striking findings was the age-related shift in lesion pigmentation. Younger patients (<20 years) predominantly presented with hyperpigmented lesions (70.0%), while older patients (>30 years) more commonly had hypopigmented lesions (60.0%). This pattern has not been extensively described in previous literature and warrants consideration of the underlying mechanisms.

Several factors may contribute to this age-related pigmentation pattern. The mechanism of pigmentation in TV differs between hyperpigmented and hypopigmented variants. Hyperpigmented lesions result from *Malassezia*-induced melanocyte stimulation and increased melanosome production, while hypopigmented lesions are thought to result from azelaic acid and other compounds produced by *Malassezia *that inhibit tyrosinase and impair melanogenesis [13]. The relative balance of these competing effects may vary with age-related changes in melanocyte function and density.

Skin pigmentation responses change with age. Younger skin typically has more active melanocytes with greater responsiveness to stimuli, potentially favoring hyperpigmentation [14]. Older skin shows decreased melanocyte density and function, which may make it more susceptible to the hypopigmenting effects of *Malassezia *metabolites [15]. Additionally, chronic sun exposure over time leads to baseline photodamage and irregular pigmentation in older adults, which may interact differently with TV-related pigmentary changes.

Anatomical distribution patterns

The finding that facial involvement was significantly more common in younger patients (35.0% vs 10-13% in older groups) is clinically important. Facial TV, while less common than truncal involvement overall, may have a greater psychosocial impact, particularly in adolescents who are typically more conscious of their appearance [16]. The higher facial involvement in younger patients may relate to increased sebaceous gland activity on the face during adolescence or different *Malassezia *colonization patterns in this age group.

The chest and upper trunk were universally the most common sites across all age groups, confirming that TV maintains its characteristic distribution pattern regardless of age. This consistency supports the role of sebum-rich areas as the primary sites for *Malassezia *overgrowth across the lifespan [1].

Sebaceous activity and age

The observed decline in greasy/seborrheic skin prevalence with increasing age (70.0% in <20 years to 40.0% in >30 years) closely parallels known patterns of sebaceous gland activity [7]. Sebum production increases dramatically at puberty under androgenic influence, remains elevated through the third decade, and then gradually declines, particularly in women after menopause [17].

This finding is particularly relevant because it suggests that while TV can occur across all age groups, the underlying predisposing factors may differ. In younger patients, active sebaceous secretion provides the lipid-rich environment that *Malassezia *requires. In older patients with reduced sebum production, other factors such as immunological changes, hormonal alterations, or environmental exposures may play a more important role in TV development [8].

Concurrent acne and shared pathophysiology

The strong inverse relationship between age and concurrent acne (70.0% in <20 years vs. 10.0% in >30 years) is expected, given acne’s well-known age distribution. However, the high co-occurrence of TV and acne in young patients (70.0%) suggests important shared pathophysiological mechanisms beyond simple sebaceous activity.

Both conditions are associated with increased sebum production, but they may also share alterations in sebum composition, follicular keratinization abnormalities, and inflammatory responses [18]. *Malassezia *species have been isolated from acne lesions and may contribute to inflammatory acne pathogenesis [19]. The high co-occurrence rate suggests that clinicians evaluating adolescents for either condition should systematically assess for the other.

Age-related comorbidities

The dramatic increase in diabetes mellitus with age (5.0% in <20 years to 85.0% in >30 years) requires careful interpretation. While this appears to show a TV-diabetes mellitus association, this finding more likely reflects selection bias (older patients in hospital-based settings typically have more comorbidities), age confounding (diabetes prevalence naturally increases with age in the general population), and referral patterns (diabetic patients may be more likely to seek medical care for skin conditions). Recent large-scale studies have actually shown that diabetes may be associated with lower, not higher, odds of TV [20]. Therefore, the high diabetes mellitus prevalence in our older TV patients should not be interpreted as evidence of causation but rather as a reflection of the comorbidity burden in older adults seeking dermatological care.

Implications for pediatric dermatology

This study contributes important data on pediatric TV, an understudied area. Our findings that children and adolescents with TV have high rates of hyperpigmented lesions (70.0%), facial involvement (35.0%), seborrheic skin (70.0%), and concurrent acne (70.0%) suggest that pediatric TV has a distinct clinical profile that may require age-specific diagnostic and therapeutic approaches. Pediatric dermatologists should be particularly alert to TV in adolescents presenting with acne and seborrheic skin, as the conditions frequently coexist [12].

Clinical applications

Our findings suggest several clinical applications. First, clinicians should maintain age-specific diagnostic awareness, considering TV across all age groups but adjusting diagnostic suspicion based on lesion characteristics. Hyperpigmented facial lesions in adolescents warrant consideration of TV, while hypopigmented truncal lesions are more typical in older adults. Second, young patients with TV should be evaluated for concurrent acne, and vice versa, given their high co-occurrence (70.0%). Third, while TV itself may not cause diabetes, older adults with TV have high rates of comorbidities that may warrant appropriate screening and management. Fourth, age-specific counseling about recurrence risk, triggering factors, and management strategies may improve outcomes, as adolescents with active sebaceous glands may need different prophylactic strategies compared to older adults. Finally, age-related factors such as medication tolerability, compliance considerations, and cosmetic concerns may influence treatment selection, with younger patients potentially more concerned about facial involvement and pigmentary changes.

Comparison with existing literature

Several studies have noted that TV predominantly affects young adults [4,5], which our data support, with 42.9% of patients aged 20-30 years. However, our finding of substantial pediatric (28.6% under 20 years) and older adult (28.6% over 30 years) representation highlights that TV is not exclusively a young adult condition. Limited prior literature exists on age-specific TV patterns. While some previous studies have examined TV epidemiology in young adults, a systematic analysis of how clinical features vary across different age groups has not been well characterized. Our study addresses this gap by characterizing how lesion type, distribution, and associated factors vary across age groups. Regarding pigmentation patterns, previous studies have described both hyperpigmented and hypopigmented TV but have not systematically examined age-related differences [13]. Our finding of age-related pigmentation shifts represents a novel contribution to the literature.

Strengths and limitations

This study has several strengths. The consecutive patient enrollment minimizes selection bias within the hospital setting. The inclusion of pediatric patients (youngest: eight years) provides rare systematic data on this age group. The comprehensive data collection on multiple clinical variables allows multifactorial analysis. The clear age-related patterns observed across multiple independent variables strengthen confidence in the findings.

However, limitations must also be acknowledged. The hospital-based sample may not be representative of community TV cases, as patients seeking hospital care may have more severe disease or more comorbidities. The single-center design limits generalizability to other geographic regions or healthcare settings. The lack of mycological confirmation (KOH examination or culture) in all cases means diagnoses were clinical, though TV has characteristic features that make clinical diagnosis reliable in experienced hands. 

The sample size, while adequate for primary analyses, limits power for some subgroup comparisons, particularly the pediatric group. Importantly, the cross-sectional design precludes the establishment of causality or temporal relationships; therefore, observed associations between age and clinical patterns cannot be definitively attributed to age-related physiological changes and may reflect confounding factors. 

Age-group categorization, while based on physiological considerations, involves arbitrary cutoffs that may not capture continuous age effects. Gender data were not systematically recorded, limiting our ability to assess potential gender-age interactions or gender-related confounding in the observed age-related patterns. Future studies should include systematic collection of gender data and multivariate analyses to account for potential confounding variables.

Future research directions

Several avenues for future investigation emerge from this study. Prospective studies with larger pediatric cohorts could better characterize childhood TV. Mechanistic studies investigating age-related differences in *Malassezia *species distribution, immune responses, and pigmentation pathways would enhance understanding of the underlying biology. Longitudinal studies following TV patients over time could assess age-related changes in clinical presentation within individuals. Community-based studies would determine if age patterns persist outside hospital settings. Intervention studies evaluating age-specific treatment protocols and their efficacy would inform clinical practice. Finally, molecular studies examining age-related differences in skin microbiome composition and sebum lipid profiles in TV patients could reveal mechanistic insights into age-dependent susceptibility.

## Conclusions

This study demonstrates that TV exhibits distinct age-related clinical patterns with important diagnostic and therapeutic implications. Younger patients predominantly present with hyperpigmented lesions, higher rates of facial involvement, seborrheic skin, and concurrent acne, while older patients more commonly show hypopigmented lesions, decreased sebaceous activity, and higher comorbidity burden. These age-specific presentations likely reflect the interaction between age-related physiological changes in sebaceous gland activity, melanocyte function, and systemic health status with *Malassezia*-induced skin changes.

Clinicians should maintain awareness of TV across all age groups while adjusting diagnostic consideration based on age-specific presentations. The high co-occurrence of TV and acne in adolescents (70.0%) suggests that evaluation for one condition should prompt assessment for the other. The substantial representation of pediatric cases in this series, along with their distinct clinical features, highlights the need for increased attention to TV in children and adolescents in both clinical practice and research. Future studies with larger pediatric cohorts, mycological confirmation, and investigation of underlying mechanisms will further clarify age-related patterns in TV and inform age-specific management strategies.
